# Near-gaze fixation promotes use of spin turns during walking: age-independent visuomotor effects with maladaptive behavioral consequences in older adults

**DOI:** 10.3389/fnagi.2026.1818850

**Published:** 2026-06-03

**Authors:** Juntaro Sakazaki, Daisuke Muroi, Takahiro Higuchi

**Affiliations:** 1 Division of Physical Therapy, Department of Rehabilitation Sciences, Faculty of Health Care Sciences, Chiba Prefectural University of Health Sciences, Chiba, Japan; 2 Department of Health Promotion Science, Tokyo Metropolitan University, Tokyo, Japan

**Keywords:** aging, anticipatory locomotor adjustments, eye-tracking, fixation, spin turn

## Abstract

**Background:**

Older adults frequently rely on spin turns while walking, a turning strategy that is biomechanically less stable than step turns and has been associated with an increased risk of falls. Previous studies have suggested that increased gaze fixation toward the area near the feet may be related to this tendency; however, the determinants of increased spin-turn use, particularly the causal role of gaze fixation, remain unclear. The present study aimed to examine whether constraining gaze fixation to the near walking location increases the use of spin turns during walking in older adults.

**Methods:**

Fifteen older adults (73.5 ± 6.3 years) and 12 younger adults (24.8 ± 3.7 years) performed a double-target stepping walking task in which the turning strategy (step turn or spin turn) was determined by foot placement relative to a second target. Gaze fixation was experimentally constrained to either a near or a far-gaze condition before stepping onto the first target. Eye movements were recorded using a mobile eye-tracking system, and whole-body kinematics were collected using a three-dimensional motion analysis system. The proportion of spin turns, frequency of pivot switching, gaze behavior, and spatiotemporal gait parameters were analyzed using generalized linear mixed models.

**Results:**

The proportion of spin turns was significantly higher under the near-gaze condition than under the far-gaze condition across both age groups, with no statistically significant difference in the magnitude of this effect between older and younger adults. Increased use of spin turns was accompanied by reduced gaze fixation on the far area during the reaching phase, shorter step length, and increased step count. Notably, older adults exhibited unnecessary pivot switching even in conditions in which a strategy change was not required. In contrast, younger adults primarily adjusted their gait patterns according to pivot-switch demands and showed less pronounced effects of gaze condition during turning strategy selection.

**Conclusion:**

These findings identify gaze fixation as a key determinant of increased spin-turn use during turning in older adults, whereby constraining gaze to the near walking surface alters visual information available for step planning and promotes maladaptive turning strategy selection.

## 1 Introduction

The risk of falls during walking increases markedly during turning (Leach et al., 2018; Robinovitch et al., 2013; Rubenstein, 2006; Tinetti et al., 1988). Among community-dwelling individuals aged ≥ 75 years, 56% experience falls while walking, with 35–40% of these falls occur during turning (Leach et al., 2018; Rubenstein, 2006; Tinetti et al., 1988). High-risk older adults turn less frequently, require more time, and exhibit greater variability in turn magnitude (Chiu et al., 2021; Leach et al., 2018), making turning a critical fall risk factor.

Two major strategies are the step turn and the spin turn. A step turn involves the contralateral (outside) limb stepping in the new direction (e.g., landing on the right foot and turning to the left), widening the base of support (BoS), facilitating the displacement of the center of mass (COM), and enabling a more stable turning motion. In contrast, a spin turn is executed by pivoting around the ipsilateral (inside) limb (e.g., landing on the right foot and turning to the right), which increases the demands on balance and joint control but allows turning while maintaining propulsion (Barrois et al., 2017; Hase and Stein, 1999; Taylor et al., 2005). Of the two strategies, spin turns are considered less stable because the whole-body COM remains outside the BoS for much of the stance phase (Fino et al., 2015; Taylor et al., 2005). Consistent with these biomechanical advantages, younger adults predominantly use step turns (Grießbach et al., 2021; Patla et al., 1991; Tillman et al., 2022). Patla et al. (1991) tasked healthy younger adults with performing 60° turns while walking and reported that more than 80% of the trials were executed as step turn (Patla et al., 1991). Tillman et al. (2022) also examined step-turn frequency in using a walking task in which healthy younger adults executed 90° turns that were either pre-planned or cued shortly before the turn (late-cued). They found that the frequency of step turns increased from 50% in the preplanned condition to 73% in the late-cued condition, indicating that younger adults preferentially shift toward step turns when faced with conditions that elevate postural instability. Overall, these findings suggest that younger adults preferentially use step turns during turning, likely to prioritize stability.

In contrast, older adults tend to use spin turns at a relatively high frequency, even under conditions associated with increased instability (Akram et al., 2010; Dixon et al., 2019; Yamada et al., 2012b). Akram et al. reported that spin turns occurred more frequently when older adults were asked to turn while walking at slower or faster walking speeds than usual (Akram et al., 2010). This suggests that, under altered walking speeds, older adults may struggle to place the contralateral limb effectively to maintain stability during turning. Dixon et al. also reported that spin turns occurred more frequently by older adults on uneven surfaces compared to flat surfaces (56% vs. 47%) and during late-cued conditions and pre-planned conditions (60% vs. 44%) (Dixon et al., 2019). This suggests that older adults appear to rely more on spin turns in situations of increased environmental demands.

The tendency of older adults to select a spin turn may be related to their increased downward gaze near their feet during walking (Chapman and Hollands, 2006; Di Fabio et al., 2003; Yamada et al., 2012a). Older adults with a history of falls have been reported to increase their fixation on the immediate walking surface, thereby limiting the gaze fixation to more distal areas of the environment (Zukowski et al., 2020). Yamada et al. (2012a) examined turning strategies using a multi-target stepping task that required participants to step onto designated targets, each of which prompted a repeated turning action during walking. They found that 63.6% of older adults at high risk of falling adopted at least one spin turn when they performed a multi-target stepping task that required participants to step onto designated targets, each of which prompted repeated turning actions during walking. This tendency was associated with an increased rate of gaze fixation on the near footfall targets. In contrast, Curzon-Jones and Hollands (2018) demonstrated that previewing the route, which prolonged fixation time on obstacles placed along the path, enhanced stepping accuracy in older adults (Curzon-Jones and Hollands, 2018). Taken together, these findings suggest that increased gaze fixation on the area near the feet may promote selection of spin turns.

While prior research has suggested a potential link between gaze fixation and turning-strategy selection, there is currently no empirical evidence based on the experimental manipulation of gaze fixation to establish a causal relationship, specifically, whether near gaze promotes the use of spin turns. To address this issue, the present study systematically examined the causal effect of gaze fixation on turning strategy selection. In particular, we investigated whether constraining gaze to a near walking-surface area increases the use of spin turns. To examine this issue, we employed an established stepping-and-turning paradigm that allows turning strategies to be identified based on foot placement patterns (Grießbach et al., 2021; Grießbach et al., 2022). We further extended this paradigm to require the use of visual information for anticipatory locomotor adjustments by introducing a second visual target positioned further along the walking path. This modification reliably elicited either step turns or spin turns, the latter being associated with greater mediolateral instability and thus representing situations in which switching to a step turn would be functionally desirable. By manipulating gaze fixation toward either near or far spatial locations, this task provides a suitable framework for testing whether gaze constraints influence the ability to flexibly select and switch turning strategies.

The present study tested three hypotheses. First, we predicted that near-gaze fixation would increase spin-turn use across both age groups; based on prior literature, we further expected this effect to be more pronounced in older adults. Second, we hypothesized that this increase in spin turns would be attributable to a reduced proportion of pivot switching under the near-gaze condition when spin turns are induced. Third, we expected that in the near-gaze condition, older adults would be unable to perform anticipatory locomotor adjustments based on visual information, resulting in a smaller change in step length.

## 2 Materials and methods

### 2.1 Participants

Fifteen older adults (eight males and seven females, mean ± SD: 73.5 ± 6.4 years old) and 12 younger adults (4 males and 8 females, 24.8 ± 3.7 years old) participated in this study. Sample size was determined using G*Power 3.1 (effect size d = 0.8, α = 0.05, power = 0.80; Heinrich-Heine-University, Düsseldorf, Germany) (Faul et al., 2007), which indicated that 12 participants were required. Considering the potential for missing gaze data in older adults, we therefore recruited 15 participants. Before performing the main experiment, participants’ details were collected. The height of each participant was measured in centimeters, and weight was measured in kilograms. We confirmed by self-report that all participants had normal or corrected-to-normal vision, no current musculoskeletal injuries, and no neurological disorders. We also assessed cognitive and mobility function in older adults; cognitive function was evaluated using the Mini-Mental State Examination (MMSE) (Folstein et al., 1975), and mobility function was assessed using the Timed Up and Go (TUG) test (Podsiadlo and Richardson, 1991). We confirmed that none of the participants had any cognitive impairment, using MMSE > 24 as a cutoff value (Folstein et al., 1975), or any mobility dysfunction, using TUG < 13.5 s as a cutoff value (Shumway-Cook et al., 2000). In addition, executive function was also evaluated using the Trail Making Test (TMT) (Ble et al., 2005).

Testing was approved by the Ethics Committee of Tokyo Metropolitan University, Japan (H6-187 and H7-032). All methods were carried out in accordance with relevant guidelines and regulations. Written informed consent was obtained from all participants in accordance with the Ethics Committee of Tokyo Metropolitan University and the Declaration of Helsinki.

### 2.2 Experimental setup

**FIGURE 1:**
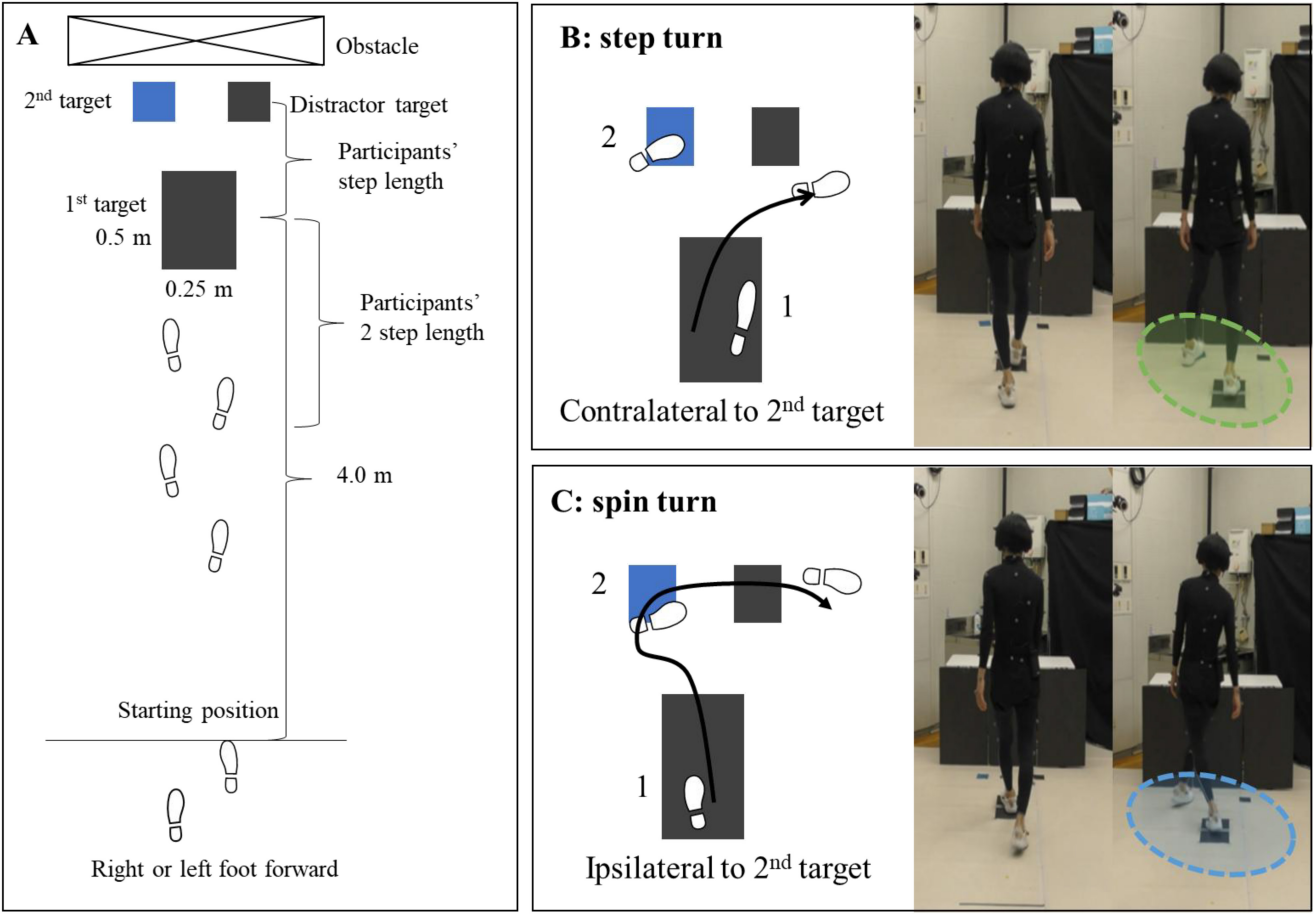
**(A)** Overview of the main experimental task. Participants initiated walking with the experimenter-specified foot, stepped continuously onto the first and second, blue-colored targets (the black target served as a dummy), and turned to the opposite side of the second target. **(B)** A step turn is elicited when the first target was stepped on with the foot contralateral to the second target. **(C)** A spin turn was elicited when the first target was stepped on with the foot ipsilateral to the second target.

The experiment was conducted in a room measuring 6.6 m × 5.6 m. The experimental setup consisted of three square targets and one obstacle positioned on a flat walking path. Among the three square targets, one was designated as a large target (first target), measuring 50 cm (length) × 25 cm (width) (Figure 1A). The remaining two were small targets (second target: 10 cm × 10 cm), colored blue and black to allow visual differentiation. The large target was placed 4.0 m from the start line, centered along the walking path. The two second targets were placed further forward, each located laterally to the left and right of the large target. The lateral distance from the center of the large target to each second target corresponded to the participant’s average step length, as measured during self-selected comfortable walking. The distance between the two small targets was fixed at 50 cm. The color assignment of the small targets on the left or right side was randomized across trials to prevent anticipatory responses. Additionally, a physical obstacle (100 cm in height and 150 cm in width) was placed 20 cm beyond the location of the small targets to discourage detours around the targets.

Body kinematics were recorded using a three-dimensional (3D) motion analysis system (Qualisys Track Manager, Qualisys, Sweden) with 15 cameras operating at a sampling frequency of 120 Hz. This system was used to track 39 infrared reflective markers that were attached to each participant’s body, based on the Vicon^®^ Plug-in Gait Model. An additional marker was attached to the front-right side of the large target.

### 2.3 Task and procedure

The experiment consisted of two components: a comfortable walking task and a double-target stepping task. First, the comfortable walking task was performed to determine the reference parameters necessary for the double-target stepping task (see details below). The comfortable walking task took place on a 5-m walkway free of obstacles. Participants stood at the start line with either their right or left foot positioned according to the experimental condition. Following a verbal instruction from the experimenter, participants were tasked with walking straight ahead at a self-selected comfortable pace. The task consisted of six trials: three trials starting with the right foot and three trials starting with the left foot. The mean step length based on each participant’s comfortable walking pace was calculated to define the target placement of the second target in the double-target stepping task. In addition, this procedure allowed us to obtain pivot foot placement data at the first target for use in the analysis of the pivot-switching behavior. The foot that first crossed the first target was defined as the pivot foot. The pivot foot that stepped on the first target during comfortable walking served as the baseline for the double-target stepping task, and pivot switching was defined as stepping with the opposite pivot foot relative to this baseline.

Second, the double-target stepping task was conducted. As in the comfortable walking task, participants began by positioning the foot specified by the experimenter (right or left) at the start line, keeping their gaze lowered while waiting for the start cue. Following the experimenter’s verbal instruction to initiate the trial, participants raised their head, walked toward the first target, and stepped on the first and second targets continuously before turning. The second target was colored blue; the remaining black target served as a dummy and was not to be stepped on. Participants were given the option to choose whether to step onto the first target with their right or left foot. The turning direction was always the opposite side of the second target, i.e., if the second target was on the left side of the two squares, then the turn direction was to the right. Based on the relationship between the participant’s choice of which foot to use to step onto the first target and the turning direction, this task configuration reliably elicited either a step turn (Figure 1B) or a spin turn (Figure 1C). Specifically, when the pivot foot stepping onto the second target was contralateral to the turning direction, a step turn was induced, whereas when it was ipsilateral to the turning direction, a spin turn was induced. Importantly, this design required participants to use visual cues from the second target to make anticipatory adjustments of foot placement prior to turning, thereby allowing them to select a safer step turn strategy by switching their pivot foot based on these visual cues in situations that would otherwise induce a spin turn.

Instructions to participants before performing the double target step walking task were as follows: “Please walk along the walkway at your normal pace and step on the first target with either your right or left foot, whichever feels more natural. You are free to adjust your stride until you reach the first target. Then, with your next step, step onto the small blue square and the turn in the opposite direction.”

**FIGURE 2:**
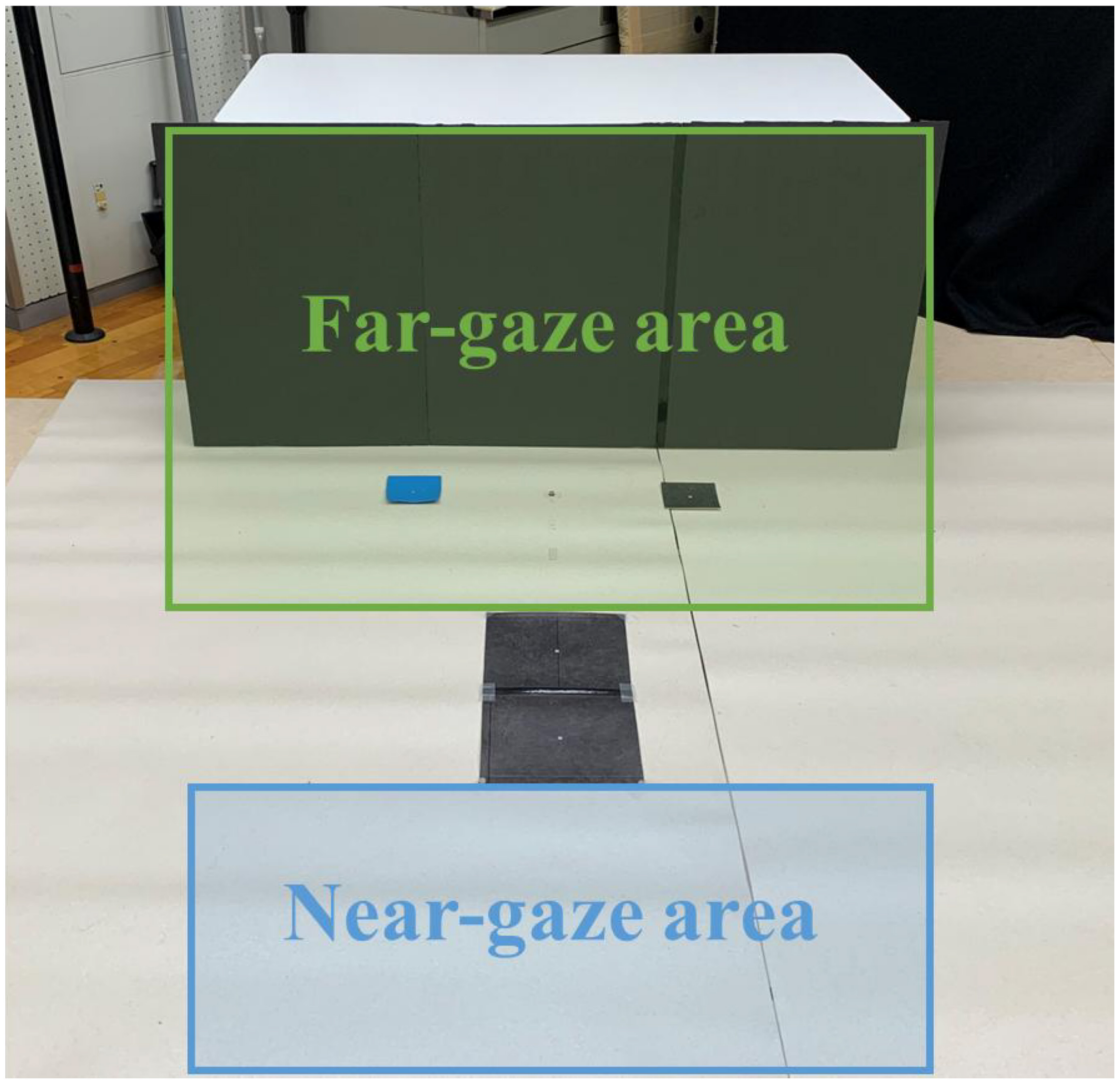
Gaze areas designated in the gaze condition. The near-gaze area extended from the start-side edge of the first target to a line located 2 strides from the center of first target, toward the start position. The far-gaze area extended from the second-target-side edge of the first target to the obstacle’s upper edge.

There were two experimental factors: pivot-switch requirement (not required vs. required) and gaze condition (near vs. far). The pivot-switch requirement factor related to whether participants needed to adjust their stride to change which foot stepped onto the first target (i.e., the pivot foot) to avoid a spin turn. A condition termed “required” indicated that participants would perform a spin turn unless they adaptively changed their foot to step onto the first target. A condition termed “not required” indicated that no change in the pivot foot was necessary. The gaze condition indicated how far participants maintained their gaze before stepping onto the first target (Figure 2). In the near-gaze condition, participants were instructed to keep their gaze on the marked near area on the walking surface. In the far-gaze condition, participants were instructed to fixate on either the second target or the obstacle, depending on the trial setup.

Ten experimental trials were performed for each of the four experimental combinations (two conditions for the pivot-switch requirement × two gaze conditions), resulting in a total of 40 trials. The pivot-switch requirement (i.e., the combination of the right or left foot forward at the start line and the blue target on either the left or right side) was randomized across trials, whereas the gaze condition was administered in a blocked format (10 trials × 2 sets per gaze condition). Four practice trials for each experimental condition were conducted prior to the experimental trials. After completion of all experimental trials, three gaze-check trials were performed, during which participants were instructed to look only at the targets (the first target and both second targets) to verify eye-tracking accuracy. To minimize fatigue effects, participants rested for at least 3 min between conditions. All participants completed the full protocol within 20–30 min.

### 2.4 Data collection

#### 2.4.1 Assessment conducted separately for approach and reaching phases

To examine the factors through which gaze fixation affects turning strategy selection, three separate analyses were conducted in addition to obtaining baseline demographic and behavioral information. All analytical measures were examined across two distinct phases: the approach phase (i.e., from the start of walking until 2 steps before reaching the first target) and the reaching phase (i.e., the final 2 steps preceding target contact). This division reflects the established dissociation between anticipatory and online locomotor control in adaptive gait (Higuchi, 2013), with the approach phase capturing predictive visual planning and the reaching phase reflecting immediate corrective responses.

#### 2.4.2 Eye-tracking data

The primary purpose of the eye-tracking data was to assess whether, during the approach phase of walking (Matthis et al., 2018), participants distributed their gaze according to the experimenter’s instructions (i.e., a manipulation check). The secondary purpose was to quantify the proportion of time participants looked at the far area during the reaching phase after the experimenter-instructed gaze-condition fixation was no longer required.

We quantified the amount of fixation directed toward each area of interest (AOI), specifically the near area, far area, target area, and other area. Fixations were defined as a gaze that remained at a given location according to the Tobii I-VT (attention) filter. This filter incorporated a velocity threshold of 100 visual degrees per second (°/s), a criterion for merging adjacent fixations (maximum angle between fixations: 0.5°; maximum time between fixations: 75 ms) and a window length of 20 ms for the I-VT fixation classifier, as set by default in Tobii Pro Lab (Tobii Technology, 2019). To quantify the duration of fixation on each AOI, the duration of the approach phase (i.e., the time required to travel from the start position to approximately 2 steps before the first target) was first calculated. Then, the duration of fixation on each AOI was calculated by dividing the total duration of fixation by the duration of the approach phase and multiplying by 100 (%) according to the following formula:


Fixation proportion for each AOI at the approach phase[%]=

$$\frac{\mathrm{duration}\,\mathrm{of}\,\mathrm{fixation}\,\mathrm{on}\,\mathrm{each}\,\mathrm{area}}{\mathrm{duration}\,\mathrm{of}\,\mathrm{the}\,\mathrm{approach}\,\mathrm{phase}}\,\text{×}\,100$$

Similarly, to analyze gaze behavior following the gaze-condition instruction, the duration of the reaching phase (i.e., the time from the end of the approach phase to stepping onto the first target) was first calculated. Then, the duration of fixation on the far area was calculated by dividing the total duration of fixation on the far area by the duration of the reaching phase and multiplying by 100 (%) according to the following formula:


Far fixation proportion at the reaching phase[%]=

$$\frac{\mathrm{duration}\,\mathrm{of}\,\mathrm{fixation}\,\mathrm{on}\,\mathrm{far}\,\mathrm{area}}{\mathrm{duration}\,\mathrm{of}\,\mathrm{the}\,\mathrm{reaching}\,\mathrm{phase}}\,\text{×}\,100$$

Trials in which the total duration of recorded gaze points did not exceed 50% of the duration of the approach phase were excluded from the analysis, following the criteria established in previous studies (Fukushima et al., 2021). A breakdown of excluded trials by age group and gaze condition is provided in Supplementary Table 1.

#### 2.4.3 Proportion of spin turns and pivot-switching assessment

To examine whether directing gaze to the near walking-surface area increased the spin-turn rate (i.e., the proportion of trials resulting in spin turns), we calculated the proportion of spin turns that occurred under each gaze condition (near vs. far). Specifically, each trial was classified as either a spin turn or a step turn based on the relationship between the foot that stepped on the first target and the side of the second target: when the pivot foot was ipsilateral to the second target, the trial was classified as a spin turn; when contralateral, as a step turn. We also examined whether participants selected the adaptive pivot-switching strategy according to the demands of the double-target stepping task. For this purpose, we recorded the frequency of pivot switching in the double-target stepping task relative to the pivot limb used in the comfortable walking task and calculated the pivot-switching rate by dividing this frequency by the 10 trials in each condition.

#### 2.4.4 Kinematics

To examine locomotor adjustments during the reaching phase, we calculated the mean step length of the 2 steps executed within that phase. Based on a previous study that investigated step length during curved and turning paths (Huxham et al., 2006), the step length was defined as the distance between two successive foot-flat positions of the foot. The step length was calculated using the following formula:


$$\mathrm{Steplength}=y\,\cos\theta =\frac{y^{2}+z^{2}-x^{2}}{2\,z}$$

where *x* indicates the absolute length of the preceding step, *y* is the absolute length of the subsequent step, and *z* is the stride distance.

To account for individual differences in natural gait characteristics, all step lengths were normalized to each participant’s mean step length measured during self-selected comfortable walking, with 100% representing the baseline. A value closer to 100% thus indicated smaller changes in step length during the reaching phase. To examine step length adjustments during the reaching phase, we analyzed the normalized step length at N (the step onto the first target) and N+1 (the step immediately before the first target). In addition, spatiotemporal gait parameters were evaluated from the start of walking to stepping on the first target, and included (i) the step count, (ii) the mean step length, (iii) the standard deviation of the step length, and (iv) the total time.

### 2.5 Statistical analysis

Descriptive statistics (mean ± SD) were calculated for participant characteristics. To compare differences between older and younger adults, independent sample *t*-tests were conducted for each variable. Where assumptions of homogeneity of variance were violated, Welch’s *t*-test was used instead. Effect sizes were computed using Cohen’s *d*, with thresholds of 0.2 (small), 0.5 (medium), and 0.8 (large) interpreted according to conventional standards.

Generalized linear mixed models (GLMMs) were used to analyze fixation proportion data, proportion of spin turns and pivot switching, and kinematics data, accounting for within-subject variability by including participant ID as a random intercept. These GLMMs were conducted separately for older and younger adults, as pooling age groups with distinct baseline motor and visual characteristics could obscure condition effects within each group; age-group comparisons within unified models were conducted only where sample sizes permitted reliable estimation, as described below. For all models, regression coefficients (β), standard errors (SE), odds ratios (OR) when applicable, 95% confidence intervals (CI), and *p*-values were reported. Statistical significance was set at *p* < 0.05, and Bonferroni correction was applied for *post-hoc* pairwise comparisons.

To verify whether gaze fixation was manipulated appropriately within each gaze condition, separate GLMMs with a normal distribution and identity link function were conducted for the near and far gaze conditions in both age groups. Gaze proportion directed toward each area of interest (near area, far area, target area, and other area) during the approach phase was the dependent variable, with area as a fixed factor.

To examine the effect of gaze area on the proportion of spin turns, a binomial GLMM with a logit link function was used. The dependent variable was the occurrence of spin turns (spin vs. step turns), and the fixed factor was area (near- vs. far-gaze condition). To directly examine whether the effect of gaze condition on spin-turn proportion differed between age groups, an additional GLMM with age group and gaze condition as fixed factors was conducted; this model constitutes the primary analysis for spin-turn proportion reported in the main text, and within-group results are provided in Supplementary Table 2.

To evaluate pivot switching, binomial GLMMs were conducted with the gaze condition (near vs. far), the pivot-switch requirement (required vs. not required), and their interaction as fixed effects. To evaluate age-related differences in condition-specific behavioral patterns, a GLMM additionally including age group as a fixed factor was conducted; full results are provided in Supplementary Table 3.

Finally, step-length adjustments, gaze behavior during the reaching phase, and spatiotemporal gait parameters were analyzed using GLMMs with the same fixed factors and interaction. Model parameters were estimated using the restricted maximum likelihood (REML). When significant main effects or interactions were observed, *post-hoc* pairwise comparisons were performed using estimated marginal means (EMMs) with Bonferroni correction.

Additionally, an exploratory GLMM examined determinants of elevated a spin-turn use in older adults during the not-required condition, using the same modeling framework as described above. The a priori power calculation was based on Cohen’s d and was not specifically calibrated for the GLMM framework; this represents a methodological limitation acknowledged in the Limitations section.

Statistical analyses were performed using IBM Statistical Package for the Social Sciences (SPSS) for Windows, version 29 (IBM Corp., Armonk, N.Y., United States). Statistical significance was set at *p* < 0.05.

## 3 Results

### 3.1 Participant characteristics

No significant differences between age groups were observed for height, step length, ΔTMT and TUG, whereas body mass and TMT Part A and Part B differed significantly (Table 1).

**TABLE 1:** Mean and standard deviation ( ± ) of anthropometric parameters and clinical tests.

Variable	Older adults (*n* = 15)	Younger adults (*n* = 12)	*P*-value	Cohen’s *d*
Age (years)	73.53 ± 6.38	24.66 ± 3.74	< 0.001	8.746
Height (cm)	161.13 ± 7.92	166.45 ± 7.51	0.088	−0.687
Weight (kg)	62.33 ± 10.19	54.58 ± 8.46	0.044	0.818
Step length (cm)	59.35 ± 8.91	64.98 ± 5.59	0.068	−0.737
MMSE (score)	29.53 ± 0.63	−	−	−
TMT-Part A (sec)	50.28 ± 25.01	32.22 ± 7.83	0.017	0.929
TMT-Part B (sec)	78.93 ± 36.97	50.9 ± 11.77	0.013	0.975
Δ TMT	28.65 ± 21.72	18.68 ± 9.48	0.125	0.572
TUG (sec)	8.77 ± 2.05	7.54 ± 1.64	0.105	0.650

MMSE, Mini-Mental State Examination; TMT, Trail Making Test. Cutoff scores, Trail Making Test Part A > 78 s; Trail Making Test Part B > 273 s (Ciolek and Lee, 2019); ΔTMT, Difference between TMT Part A and TMT Part B; TUG: Timed Up and Go Test. Cutoff time: 13.5 s, commonly used to indicate increased fall risk (Shumway-Cook et al., 2000).

### 3.2 Eye tracking data

The GLMMs for fixation proportion during the approach phase revealed significant fixed effects of gaze-location condition in both older and younger adults under both gaze conditions (all *p* < 0.001).

**FIGURE 3:**
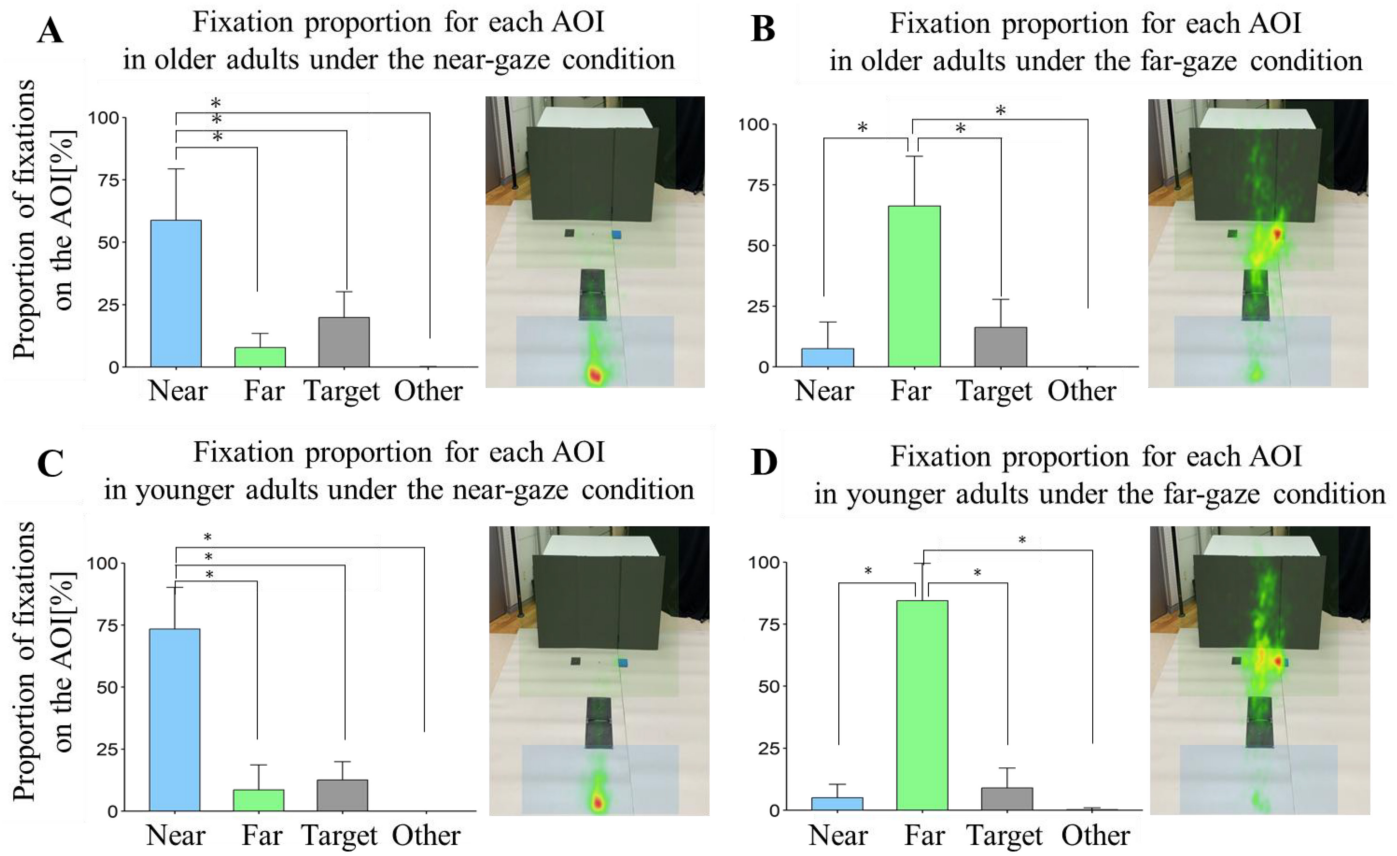
Mean ( ± standard deviation) fixation proportion for each AOI during the approach phase. An asterisk (*) indicates a significant difference (*p* < 0.05). Heat maps of fixation rates per AOI across conditions for each participant. The maps are overlaid on the stimulus images, with hotter colors (e.g., red) indicating targets receiving higher fixation rates and cooler colors (e.g., blue/green) indicating lower rates. The data are averaged across all participants. **(A)** Near-gaze condition in older adults; **(B)** far-gaze condition in older adults; **(C)** near-gaze condition in younger adults; **(D)** far-gaze condition in younger adults. Both groups exhibited experimentally manipulated differences in gaze fixation, confirming successful experimental manipulation.

In older adults under the near gaze condition, compared with the near area, the far area [β = -54.49, SE = 17.58, *p* = 0.002, 95% CI (−88.99, −20.00)], the target area [β = -44.38, SE = 17.58, *p* = 0.012, 95% CI (−78.88, −9.89)], and the other area [β = -62.21, SE = 17.58, *p* < 0.001, 95% CI (−96.70, −27.72)] all showed significantly lower gaze proportions (Figure 3A). Similarly, under the far-gaze condition, compared with the far area, the near area [β = -59.25, SE = 21.33, *p* = 0.006, 95% CI (−101.16, −17.39)], the target area [β = -50.95, SE = 21.33, *p* = 0.017, 95% CI (−92.84, −9.09)], and other areas [β = -66.75, SE = 21.33, *p* = 0.002, 95% CI (−108.67, −24.89)] all showed significantly lower gaze proportions (Figure 3B).

In younger adults, under the near-gaze condition, compared with the near area, the far area [β = -64.32, SE = 1.42, *p* < 0.001, 95% CI (−67.10, −61.54)], the target area [β = -60.30, SE = 1.42, *p* < 0.001, 95% CI (−63.08, −57.52)], and other areas [β = -72.82, SE = 1.42, *p* < 0.001, 95% CI (−75.60, −70.04)] all showed significantly lower gaze proportions (Figure 3C). Similarly, under the far-gaze condition, compared with the far area, the near area [β = -79.61, SE = 1.23, *p* < 0.001, 95% CI (−82.02, −77.19)], the target area [β = -75.41, SE = 1.23, *p* < 0.001, 95% CI (−77.82, −72.99)], and other areas [β = -84.20, SE = 1.23, *p* < 0.001, 95% CI (−86.61, −81.78)] all showed significantly lower gaze proportions (Figure 3D).

These results confirm that the experimental manipulation was successfully implemented, such that gaze fixation was appropriately directed to the near area in the near-gaze condition and to the far area in the far-gaze condition.

**FIGURE 4:**
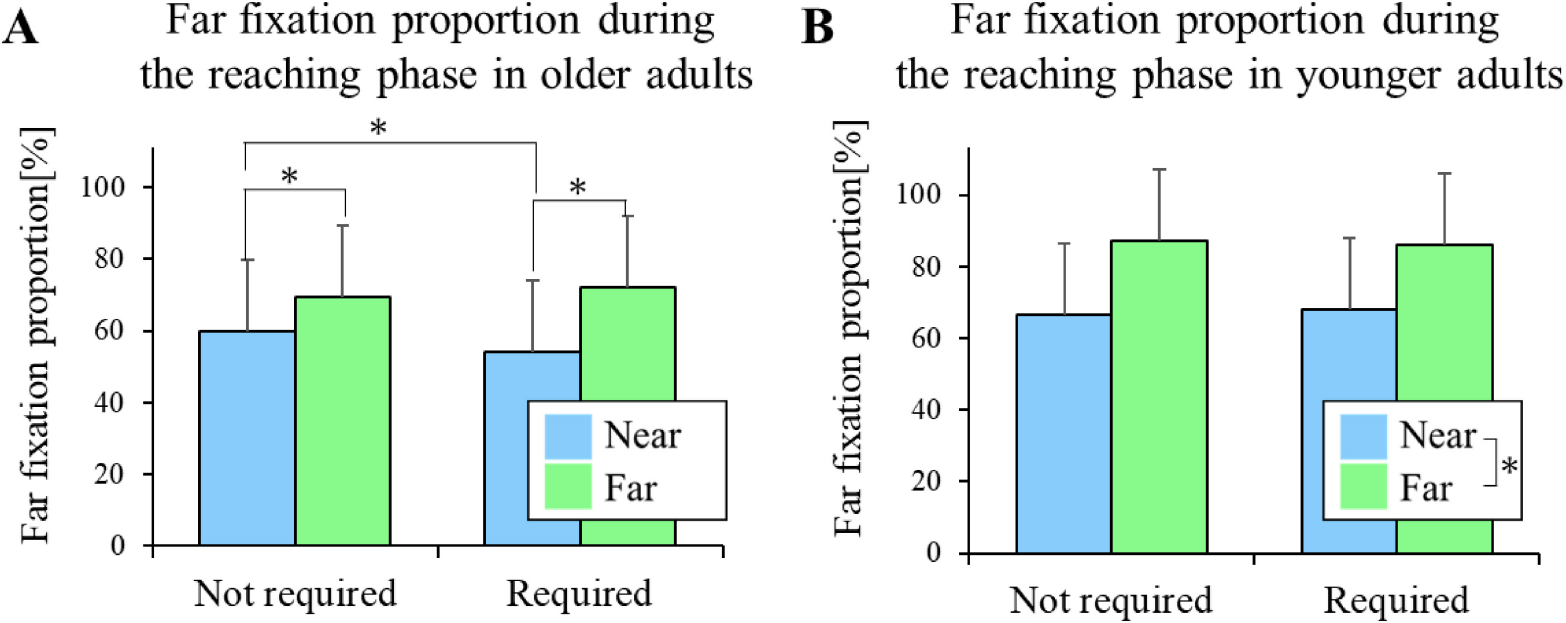
Far fixation proportion during the reaching phase. Bars represent estimated marginal means from the GLMM, and error bars indicate standard errors. Asterisks (*) denote significant differences (*p* < 0.05). The data is averaged across all participants. **(A)** Older adults. **(B)** Younger adults.

The results of the GLMM for the far fixation proportion during the reaching phase in older adults are shown in Figure 4A. The analysis revealed a fixed effect of the pivot-switch requirement [β = –5.70, *SE* = 2.89, *t* = –1.97, *p* = 0.049, 95% *CI* (−-11.36, –0.03)] and the gaze-location condition [β = 9.81, *SE* = 2.89, *t* = 3.39, *p* = 0.001, 95% *CI* (4.13, 15.49)]. Importantly, the interaction between the pivot-switch requirement and the gaze condition was also significant [β = 8.09, *SE* = 4.10, *t* = 1.97, *p* = 0.049, 95% *CI* (0.02, 16.15)]. *Post-hoc* analyses revealed that the far fixation proportion during the reaching phase was significantly smaller in the required condition than in the not-required condition under the near-gaze condition (*p* = 0.049). In addition, the far fixation proportion during the reaching phase was smaller in the near-gaze condition compared with the far-gaze condition in both the required and not-required conditions (both *p* < 0.001).

The results of the GLMM for the far fixation proportion during the reaching phase in younger adults are shown in Figure 4B. The analysis revealed a significant main effect of the gaze condition [β = 20.55, *SE* = 2.60, *t* = 7.89, *p* < 0.001, 95% *CI* (15.44, 25.67)], indicating that far areas elicited a larger proportion of far-gaze location compared with near-gaze location. In contrast, the main effect of the pivot-switch requirement [β = 1.60, *SE* = 2.60, *t* = 0.62, *p* = 0.538, 95% *CI* (−-3.50, 6.71)] and the pivot-switch requirement × the gaze-condition interaction [β = –2.56, *SE* = 3.70, *t* = –0.69, *p* = 0.490, 95% *CI* (−-9.82, 4.71)] were not significant.

Taken together, these results suggest that gaze behavior in older adults was sensitive to both gaze condition and pivot-switch requirement, while younger adults’ gaze was predominantly guided by gaze condition alone.

### 3.3 Proportion of spin turns and frequency of pivot switching

A GLMM with age group and gaze condition as fixed factors revealed a significant main effect of gaze condition [β = 0.360, SE = 0.174, *t* = 2.069, *p* = 0.039, OR = 1.433, 95% CI (1.019, 2.017)], indicating that near-gaze fixation increased spin-turn use across both age groups. The main effect of age group (*p* = 0.417) and the age group × gaze condition interaction [β = 0.032, SE = 0.268, *t* = 0.120, *p* = 0.904, OR = 1.033, 95% CI (0.610, 1.748)] were not significant, indicating that the magnitude of the gaze-condition effect was statistically equivalent between older and younger adults. Within-group analyses are provided in Supplementary Table 2 for reference.

A GLMM with age group, gaze condition, and pivot-switch requirement as fixed factors was conducted to evaluate age-related differences in condition-specific behavioral patterns. Although significant two-way interactions were observed for age group × pivot-switch requirement [β = -0.919, SE = 0.376, *t* = -2.443, *p* = 0.015, 95% CI (−1.658, −0.181)], age group × gaze condition [β = -0.678, SE = 0.251, *t* = -2.697, *p* = 0.007, 95% CI (−1.171, −0.185)], and pivot-switch requirement × gaze condition [β = 0.828, SE = 0.262, *t* = 3.163, *p* = 0.002, 95% CI (0.314, 1.342)], the three-way interaction was not significant [β = 0.280, SE = 0.466, *t* = 0.601, *p* = 0.548, 95% CI (−0.634, 1.195)]. Full results are provided in Supplementary Table 3.

**FIGURE 5:**
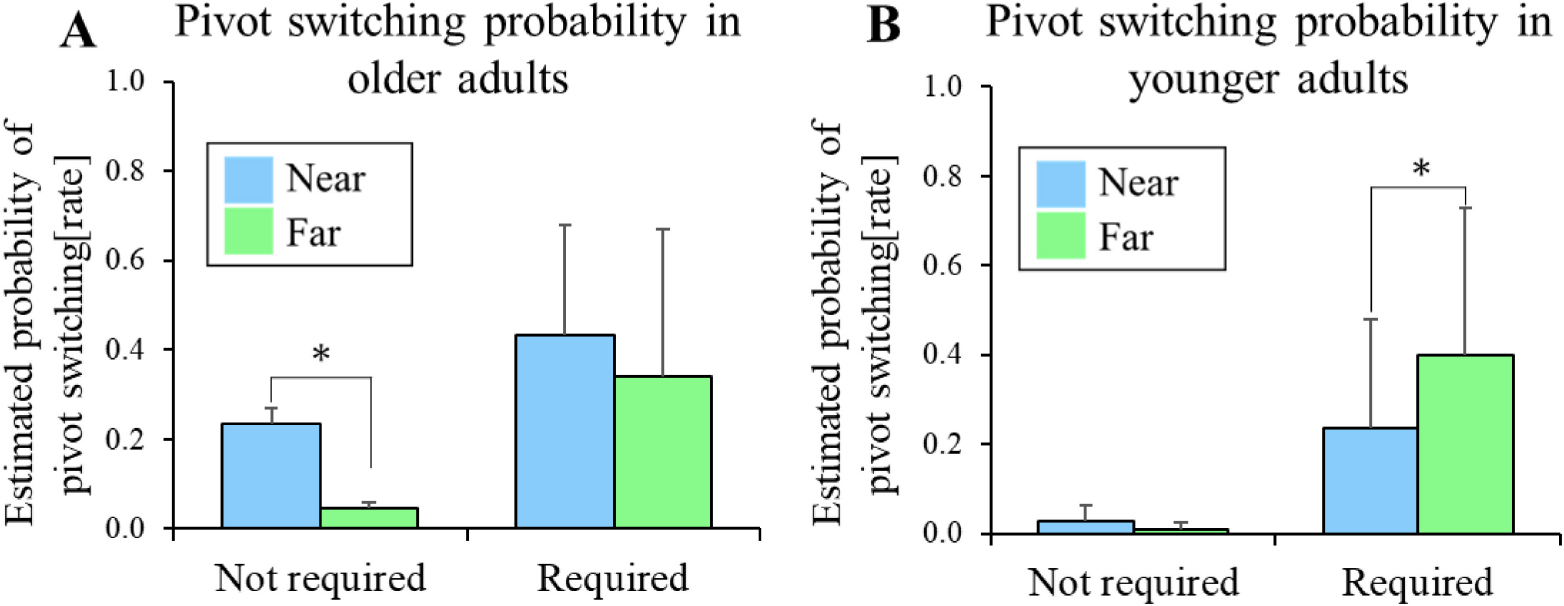
Estimated probability of turn switching derived from the GLMM. The y-axis represents the estimated probability of pivot switching. Error bars represent ± SE. Asterisks indicate significant differences (*p* < 0.05). **(A)** Older adults. **(B)** Younger adults.

Within-group analyses revealed the following condition-specific patterns. The results of the GLMM for the frequency of pivot switching in older adults are shown in Figure 5A. The analysis revealed a significant fixed effect on the pivot-switch requirement [β = 0.92, *SE* = 0.28, *p* = 0.001, *OR* = 2.51, 95% *CI* (1.45, 4.34)], and a significant fixed effect of the gaze condition [β = –1.87, *SE* = 0.37, *p* < 0.001, *OR* = 0.15, 95% *CI* (0.07, 0.32)]. In addition, the pivot-switch requirement × the gaze condition interaction was significant [β = 1.48, *SE* = 0.46, *p* = 0.001, *OR* = 4.39, 95% *CI* (1.77, 10.87)]. Pairwise comparisons (Bonferroni-corrected) further revealed that within the required condition, switching frequency did not significantly differ between near- and far-gaze conditions (*p* = 0.156). In contrast, within the not-required condition, switching frequency was significantly higher under the near-gaze condition than under the far-gaze condition (*p* < 0.001). When comparing the required and not-required conditions for each gaze condition, the switching frequency was significantly higher under the required condition than in the not-required condition under both far (*p* < 0.001) and near (*p* = 0.001) gaze conditions. These results suggest that older adults tend to perform unnecessary pivot switching even in the not-required condition, in which a strategy change is not required.

The results of the GLMM for the frequency of pivot switching in younger adults are shown in Figure 5B. The analysis revealed a significant fixed effect of the pivot-switch requirement [β = 2.40, *SE* = 0.46, *p* < 0.001, *OR* = 11.07, 95% *CI* (4.46, 27.47)]. The main effect of the gaze condition was not significant [β = –1.08, *SE* = 0.71, *p* = 0.126, *OR* = 0.34, 95% *CI* (0.08, 1.36)]. In addition, the pivot-switch requirement × the gaze condition interaction was significant [β = 1.86, *SE* = 0.78, *p* = 0.018, *OR* = 6.45, 95% *CI* (1.38, 30.19)]. Pairwise comparisons revealed that the required condition showed significantly higher switching frequency than the not-required condition under both the far-gaze condition (*p* < 0.001) and the near-gaze condition (*p* < 0.001). For the required condition, switching frequency was significantly higher under the near-gaze condition than the far-gaze condition (*p* = 0.022). In contrast, for the not-required condition, no significant difference was observed between the near- and far-gaze conditions (*p* = 0.126).

### 3.4 Kinematics

The results of the GLMM analyses of gait parameters by age group are shown in Table 2. In older adults, the gaze-location factor showed a significant fixed effect on multiple gait parameters. Specifically, during the approach phase, step length at the first target (N) was shorter in the near-gaze condition than in the far-gaze condition. Across the entire trial, the near-gaze condition was also associated with a greater total number of steps, shorter average step length, and a tendency toward longer completion time. In contrast, the pivot-switch factor did not exhibit any significant fixed effects on these measures, nor were there significant interactions between the pivot-switch and the gaze location.

**TABLE 2:** Results of the generalized linear mixed model (GLMM) analysis of gait parameters by age group.

Variable	Group	Pivot-switch effect	Gaze-location effect	Interaction effect
		β [95%CI]	β [95%CI]	β [95%CI]
Reaching phase				
N	OA	−0.1 [−3.16, 2.96]	4.38 [1.33, 7.43]*	2.2 [−6.07, 2.58]
	YA	−7.08 [−9.87, −4.29]*	−0.23 [−3.02, 2.56]	2.01 [−1.73, 6.16]
N+1	OA	−1.55 [−4.72, 1.61]	2.28 [−0.87, 5.45]	2.28 [−5.71, 3.25]
	YA	−3.8 [−6.1, −1.5]*	1.13 [−1.16, 3.43]	1.65 [−1.52, 4.98]
Approach and reaching phase				
Step counts	OA	0 [−0.11, 0.09]	−0.25 [−0.36, −0.15]*	0.07 [−0.11, 0.18]
	YA	0.15 [0.04, 0.27]*	−0.21 [−0.33, −0.09]*	0.08 [−0.12, 0.2]
Normalized step length	OA	0 [−1.48, 1.46]	2.54 [1.07, 4.01]*	1.05 [−2.51, 1.64]
	YA	−1.84 [−3.16, −0.52]*	1.11 [−0.2, 2.44]	0.95 [−1.06, 2.67]
Step length SD	OA	0 [−0.01, 0]	0 [−0.01, 0]	0 [−0.01, 0.01]
	YA	0 [0, 0]	0 [−0.01, 0]	0 [0, 0.01]
Total time	OA	0 [−0.04, 0.04]	−0.14 [−0.18, −0.09]*	0.03 [−0.03, 0.09]
	YA	0.04 [0, 0.09]	−0.1 [−0.15, −0.05]*	0.03 [−0.05, 0.09]

OA, older adults; YA, younger adults. β, Regression coefficient; 95% CI, 95% confidence interval; *p < 0.05. *N* = Step length at the first target; N+1 = Step length one step before the first target.

In younger adults, a significant fixed effect of the pivot-switch factor was observed. During the approach phase, step length at the first target (N) and one step before the first target (N+1) was shortened in the required condition. Throughout the task, this was accompanied by an increase in the number of steps and a general shortening of step length in the required condition. In addition, the main effect of the gaze location was observed, characterized by an increase in the total number of steps and longer task completion time throughout the task. No interactions between the pivot-switch and the gaze location were observed.

Taken together, these findings indicate that older adults primarily modified their gait patterns in response to the gaze-location factor, whereas younger adults adapted their gait patterns according to the pivot-switch factor.

### 3.5 Determinants of elevated pivot switching in older adults during the not-required condition

As an additional analysis, a GLMM was conducted to identify factors associated with pivot turn switching in older adults during the not-required condition. The dependent variable was the occurrence of switching (0 = no switch, 1 = switch), modeled using a binomial distribution with a logit link function. The fixed effects included the far-gaze proportion during the reaching phase, N, N+1, step counts, normalized step length, standard deviation of step length, and total time, with participant ID included as a random intercept.

The GLMM revealed significant fixed effects of the far-gaze proportion during the reaching phase and N. Specifically, the far-gaze proportion during the reaching phase was negatively associated with switching [β = -0.016, *SE* = 0.0066, *t* = -2.475, *p* = 0.014, *OR* = 0.984, *95% CI* (0.971, 0.997)]. Likewise, the step length at the first target (N) showed a significant negative association [β = -0.072, *SE* = 0.0160, *t* = -4.503, *p* < 0.001, *OR* = 0.931, *95% CI* (0.902, 0.960)]. These findings indicate that a higher proportion of gaze directed toward the far area during the reaching phase and a smaller the step length at the first target were both associated with a reduced likelihood of spin turn switching. In contrast, none of the other predictors (step length one step before the first target (N+1), step count, normalized step length, standard deviation of step length, or total time) showed significant effects (all *p* > 0.05).

## 4 Discussion

The present study examined whether constraining gaze fixation to the near walking surface influences turning strategy selection, with particular focus on the mechanisms underlying increased spin-turn use in older adults. Gaze fixation was appropriately directed to the designated area during the approach phase in both age groups (Figure 3), confirming successful gaze manipulation. Our first hypothesis, that the near gaze-location condition increases spin turns, was supported in both age groups. The second hypothesis, predicting reduced pivot switching rates under the near-gaze condition in the required condition, was supported in younger adults but not in older adults. The third hypothesis, that the near-gaze condition would result in a smaller rate of change in step length, was not supported in either age group. Nevertheless, whereas younger adults demonstrated adaptive strategies by adjusting the step length and switching the pivot foot in response to task demands, older adults exhibited maladaptive strategies, uniformly shortening the step length in the near-gaze condition and showing unnecessary pivot switching even in the not-required condition (Table 2). These findings suggest that gaze fixation toward the near walking surface during the approach phase leads to a conservative strategy that promotes spin-turn use in older adults.

The first hypothesis was partially supported. A GLMM with age group and gaze condition as fixed factors confirmed that near-gaze fixation increases spin-turn use across both age groups (*p* = 0.039); however, the age group × gaze condition interaction was not significant (*p* = 0.904), indicating that the hypothesized age-specific amplification of this effect was not statistically supported. These results suggest that near-gaze fixation operates as a general visuomotor mechanism that increases spin-turn use regardless of age. Directionally, older adults showed a significant within-group effect (*p* = 0.040) while younger adults did not (*p* = 0.053), a pattern consistent with our original hypothesis, but one that should not be interpreted as a statistically reliable between-group difference. This increase is consistent with the findings of Dixon et al., who reported a similar 1.31-fold increase when comparing uneven and flat surfaces, suggesting that environmental and behavioral factors are similarly associated with increased spin-turn use (Dixon et al., 2019). In the present study, spin turns represent a riskier strategy than step turns, involving closer foot-to-foot distances and greater mediolateral balance constraints (Taylor et al., 2005). The finding that experimentally constraining gaze fixation to the near area increased the use of this more hazardous turning strategy provides evidence for causal contribution of gaze fixation to maladaptive strategy selection. Turning imposes greater frontal-plane balance demands than straight walking (Tillman et al., 2022), particularly under conditions that reduce predictability. Dixon et al. demonstrated that late-cued conditions increase spin-turn use (Dixon et al., 2019), while Akram et al. reported increased spin-turn preference at non-comfortable speeds (Akram et al., 2010), suggesting that older adults adopt spin turns under challenging conditions that compromise movement control. In the present study, constraining gaze fixation to the near area may have similarly limited environmental scanning and predictive visual information acquisition, creating balance-control demands analogous to late-cued conditions. This interpretation is supported by Yamada et al., who showed that high-risk older adults exhibited increased fixation to areas near the feet and more frequent spin turns during multi-target stepping (Yamada et al., 2012a). These findings suggest that spin-turn use is attributable to gaze fixation patterns toward areas near the feet, which may impair the ability to plan safer turning strategies regardless of age.

This decline in strategy planning ability due to gaze fixation was reflected in kinematics parameters, including pivot switching and step-length adjustments. Younger adults showed results partially consistent with our predictions, whereas older adults exhibited overly conservative strategies, resulting in pivot switching even in the not-required condition (Figure 5A). Older adults reduced the step length in response to near-gaze fixation and increased step counts (Table 2). This reduction can be interpreted as compromised feedforward control based on distal visual information. Feedforward control is essential for efficient locomotion (Matthis et al., 2017), and its decline may deteriorate gait efficiency. Ellmers and Young demonstrated that anxiety increases proximal fixation and reduces distal environmental scanning, thereby limiting visual information acquisition for future step planning and providing a potential explanation for the reduced feedforward control observed in older adults (Ellmers and Young, 2019). High-risk older adults prioritize future step planning over accurate execution of ongoing steps (Chapman and Hollands, 2007) and exhibit altered gaze distribution, resulting in insufficient distal information acquisition (Young and Hollands, 2010). In addition, altered visual search characteristics in older adults have been shown to induce conservative gait strategies with shortened step length and reduced speed (Jia et al., 2025), which is consistent with our findings. This tendency was more pronounced in the near-gaze condition, and excessive pivot switching can be interpreted as a consequence of excessive attention to future planning, resulting in insufficient visual information for current stepping. These findings suggest that near-gaze fixation reduces feedforward control in older adults and induces overly conservative motor control strategies, eliciting maladaptive motor parameter adjustments, including unnecessary pivot switching and step-length reduction.

These results suggest that experimental manipulation of gaze constraints may enable assessment of functional decline in feedforward control and disruptions in adaptive turning strategies. Visuomotor control is based on anticipation of future events (Higuchi, 2013; McFadyen and Carnahan, 1997), with walkers directing gaze fixation toward distal areas several steps ahead to pre-plan motor actions (Matthis et al., 2018; Patla and Vickers, 2003). Predictive feedforward control and swing-phase online control both support adaptive locomotion (Cates and Gordon, 2022; Chapman and Hollands, 2006). During turning, predictive gaze fixation enables advance strategy selection (Hollands et al., 2002). In the present study, constraining distal gaze fixation in the near-gaze condition disrupted feedforward control, increasing spin-turn use in both age groups (Hypothesis 1). Younger adults maintained their pivot strategy via flexible online control switching (Hypothesis 2), whereas older adults had difficulty switching strategies due to reduced visuomotor adjustment ability (Chapman and Hollands, 2006), leading to overly conservative strategy selection (Hypothesis 3). These findings suggest that the eye-tracking system employed here can be applied as an experimental manipulation to induce and evaluate functional decline in feedforward control caused by gaze constraints, providing a novel methodological approach for detecting disruptions in adaptive turning strategies among older adults.

The present findings can be interpreted within the affordance competition hypothesis framework (Cisek, 2007), offering both theoretical and practical implications. According to this hypothesis, walkers perceive multiple action possibilities (affordances) based on visual information, and select actions according to motor costs and goal achievement likelihood (Gordon et al., 2021). Previous studies have shown that motor costs influence action selection during walking (Grießbach et al., 2021; Grießbach et al., 2022), and gaze fixation patterns change under high motor-cost conditions (Domínguez-Zamora and Marigold, 2019). In the present study, the far-gaze condition likely enabled advance preparation through distal visual information, promoting step-turn selection with lower motor costs. Conversely, the near-gaze condition restricted visual information, impairing advance preparation and leaving insufficient time for step-turn decisions. As reduced predictive gaze fixation impairs walking performance in older adults (Ellmers et al., 2020; Uiga et al., 2015), the proximal gaze condition experimentally reproduced this pattern. Theoretically, these findings demonstrate the causal influence of gaze constraints on turning strategy selection within the affordance competition framework. Practically, given that gaze strategy interventions improve stepping accuracy in older adults (Curzon-Jones and Hollands, 2018; Young and Hollands, 2010), these findings suggest the potential for fall prevention through visuomotor control interventions.

Although the experimental task was specifically designed to allow causal inference regarding the influence of gaze fixation on turning strategy selection, this level of experimental control necessarily reduces correspondence with naturalistic turning behavior in daily life. In everyday walking, turns are self-initiated, vary continuously in angle and speed, and occur within complex multimodal environments. Nonetheless, the near-gaze constraint employed in this study experimentally reproduces a gaze pattern documented in older adults with elevated fall risk during free walking (Yamada et al., 2012a; Zukowski et al., 2020), lending translational relevance to the present findings. Future studies employing mobile eye-tracking during unstructured community walking would be valuable for determining whether the gaze–strategy relationships observed here generalize to naturalistic turning contexts.

This study had four limitations. First, participants in this study were older adults with good physical and cognitive function (TUG: 8.77 ± 2.05 s, MMSE: 29.53 ± 0.63 points, no fall history). Therefore, the applicability of our findings to older adults at high risk of falling (Yamada et al., 2012a) or individuals with movement disorders such as Parkinson’s disease, who exhibit difficulties in step length adjustment (Conradsson et al., 2017), remains unclear. Future studies should include more diverse populations across different functional levels and clinical conditions to establish the generalizability of gaze constraints on turning strategy selection.

Second, the frequency of spin turns observed in this study (near-gaze condition: 42%, far-gaze condition: 34%) was lower than that reported in previous studies using similar pre-planned turning tasks (50–56%) (Dixon et al., 2019; Tillman et al., 2022). This may reflect task-specific avoidance of unstable turning situations by participants. As discussed above, the structured nature of the experimental task also limits the generalizability of the present findings to naturalistic turning behavior in daily life.

Third, the a priori power calculation employed in this study was based on Cohen’s d and was not specifically calibrated for the GLMM framework used in the primary analyses; GLMM-specific power estimation, such as simulation-based approaches, should be employed in future studies. Furthermore, although a GLMM with age group and gaze condition as fixed factors confirmed that near-gaze fixation increases spin-turn use across both age groups, the age group × gaze condition interaction was not significant (*p* = 0.904), indicating that the hypothesized age-specific amplification of this effect could not be statistically established in the current sample. Similarly, the three-way interaction for pivot-switching frequency was not significant (*p* = 0.548), and the constraints imposed by the current sample sizes (older adults: *n* = 15; younger adults: *n* = 12) precluded extension of unified age-group models to the remaining outcomes. Future studies with larger, balanced samples are needed to formally evaluate age-specific effects in visuomotor control during turning across all outcome measures.

Fourth, although we collected data from younger adults, the primary focus of this study was to elucidate the mechanisms underlying increased spin-turn use in older adults, and the analytical framework was designed accordingly. While the age-group models conducted in the present study did not reveal a statistically reliable between-group difference in the gaze-condition effect on spin-turn proportion, age-related changes in turning behavior have been documented across multiple domains (Madrid et al., 2023; Nakamura et al., 2023; Sakazaki et al., 2025). Future research incorporating larger samples and a more balanced between-group design would enable clearer identification of age-specific changes in visuomotor control during turning.

## 5 Conclusion

Constraining gaze fixation toward the near walking surface during the approach phase increased spin-turn use across both age groups and reduced gaze distribution to far areas during the reaching phase; older adults additionally exhibited maladaptive behavioral patterns including unnecessary pivot switching and step-length reduction. Our findings suggest that gaze fixation toward the near walking surface influences visuomotor control processes and promotes conservative strategy selection in older adults. The eye-tracking system employed here provides a novel methodological approach for evaluating gaze-related disruptions in adaptive turning strategies among older adults.

## Data Availability

The raw data supporting the conclusions of this article will be made available by the authors upon reasonable request.
