# Supplementary material for: Near-gaze fixation promotes use of spin turns during walking: age-independent visuomotor effects with maladaptive behavioral consequences in older adults
Source: Front Aging Neurosci. 2026 Jun 3;18:1818850. doi: 10.3389/fnagi.2026.1818850 (PMC13273758; doi:10.3389/fnagi.2026.1818850)
Supplement: Supplementary file 1 [file Data_Sheet_1.docx]

Supplementary Material

# Supplementary Tables

**Supplementary Table 1. Breakdown of excluded trials by age group and gaze condition (number; percentage of total trials)**

Trials in which the total duration of recorded gaze points did not exceed 50% of the duration of the approach phase were excluded from the eye-tracking analyses. The table below presents the number and percentage of excluded trials by age group and gaze condition. Exclusion rates were higher in older adults, particularly in the near-gaze condition. This pattern was primarily attributable to eyelid descent during sustained downward gaze, which causes clipping of the pupil and loss of infrared illuminator reflections, thereby interrupting gaze tracking (50). This phenomenon is a recognized limitation of wearable infrared eye trackers during downward gaze tasks (50, 51), and may be exacerbated in older adults due to age-related changes in eyelid position. The differences in exclusion rates across conditions were small and are unlikely to have systematically biased the reported findings.

|  | **Near-gaze condition** | **Far-gaze condition** | **Total** |
| --- | --- | --- | --- |
| Older adults (n = 15; 300 trials/condition) | 34 (11.33%) | 15 (5.00%) | 49 (8.17%) |
| Younger adults (n = 12; 240 trials/condition) | 0 (0.00%) | 1 (0.41%) | 1 (0.21%) |

*Note. Values represent the number of excluded trials and the corresponding percentage of total trials. Denominators: older adults = 300 trials per condition (15 participants × 20 trials); younger adults = 240 trials per condition (12 participants × 20 trials). Total column reflects exclusions across both gaze conditions combined (older adults: 600 trials total; younger adults: 480 trials total).*

**Supplementary Table 2. Within-group GLMM results for spin-turn proportion**

Results of the within-group generalized linear mixed models (GLMMs) for the proportion of spin turns in older and younger adults separately. Each model included gaze condition (near vs. far) as the sole fixed factor, with participant ID as a random intercept and a binomial distribution with logit link function. The reference category for gaze condition is the far-gaze condition. The primary analysis for spin-turn proportion reported in the main text is a GLMM including age group and gaze condition as fixed factors; these within-group results are provided for reference.

| **Group** | **β** | **SE** | **t** | **p** | **OR** | **95% CI** |
| --- | --- | --- | --- | --- | --- | --- |
| Older adults (gaze condition: near vs. far) | −0.356 | 0.173 | −2.057 | **.040** | 0.701 | [0.499, 0.984] |
| Younger adults (gaze condition: near vs. far) | −0.399 | 0.206 | −1.939 | .053 | 0.671 | [0.448, 1.005] |

*Note. β = regression coefficient; SE = standard error; OR = odds ratio; 95% CI = 95% confidence interval. Bold p-values indicate statistical significance (p < .05). The p-value of .053 for younger adults does not constitute a statistically significant effect and should not be interpreted as a trend toward significance.*

**Supplementary Table 3. GLMM for pivot-switching frequency with age group, gaze condition, and pivot-switch requirement as fixed factors**

Results of a GLMM for pivot-switching frequency including age group (OA vs. YA), gaze condition (near vs. far), and pivot-switch requirement (step vs. spin) as fixed factors, with participant ID as a random intercept and a binomial distribution with logit link function. This model was conducted to evaluate age-related differences in condition-specific behavioral patterns. Although significant two-way interactions were observed, the three-way interaction (age group × gaze condition × pivot-switch requirement) was not significant (p = .548), indicating that the age-specific pattern of pivot-switching across conditions could not be formally established in this model. Condition-specific analyses conducted separately for each age group are reported in the main manuscript.

| **Model term** | **β** | **SE** | **t** | **p** | **95% CI** |
| --- | --- | --- | --- | --- | --- |
| Intercept | −0.390 | 0.898 | −0.435 | .664 | [−2.152, 1.371] |
| Age group (YA vs. OA) | 0.163 | 0.410 | 0.398 | .690 | [−0.641, 0.968] |
| Pivot-switch requirement (step vs. spin) | −1.393 | 0.205 | −6.809 | **<.001** | [−1.795, −0.992] |
| Gaze condition (near vs. far) | 0.237 | 0.163 | 1.448 | .148 | [−0.084, 0.557] |
| Age × Pivot-switch requirement | −0.919 | 0.376 | −2.443 | **.015** | [−1.658, −0.181] |
| Age × Gaze condition | −0.678 | 0.251 | −2.697 | **.007** | [−1.171, −0.185] |
| Pivot-switch requirement × Gaze condition | 0.828 | 0.262 | 3.163 | **.002** | [0.314, 1.342] |
| Age × Pivot-switch requirement × Gaze condition (3-way interaction) | 0.280 | 0.466 | 0.601 | .548 | [−0.634, 1.195] |

*Note. β = regression coefficient; SE = standard error; 95% CI = 95% confidence interval. OA = older adults; YA = younger adults. Reference categories: age group = OA; pivot-switch requirement = spin (required); gaze condition = far-gaze. Bold p-values indicate statistical significance (p < .05). Significant two-way interactions were observed for age group × pivot-switch requirement (p = .015), age group × gaze condition (p = .007), and pivot-switch requirement × gaze condition (p = .002).*

*References cited in this Supplementary Material are included in the reference list of the main manuscript.*
